# Rebuilding transformation strategies in post-Ebola epidemics in Africa

**DOI:** 10.1186/s40249-017-0278-2

**Published:** 2017-05-10

**Authors:** Ernest Tambo, Chryseis F. Chengho, Chidiebere E. Ugwu, Isatta Wurie, Jeannetta K. Jonhson, Jeanne Y. Ngogang

**Affiliations:** 1 Higher Institute of Health Sciences, Université des Montagnes, Bangangte, Cameroon; 2 Africa Disease Intelligence and Surveillance, Communication and Response (Africa DISCoR) Institute, Yaoundé, Cameroon; 3 Department of Health and Life Sciences, University of Coventry, Coventry, Leicester, UK; 4 Faculty of Basic Medical Sciences, Department of Human Biochemistry, NnamdiAzikiwe University Awka, Nnewi Campus, Awka, Nigeria; 5 Department of Chemical Pathology, College of Medicine and Allied Health Sciences, University of Sierra Leone, Freetown, Sierra Leone; 6 Public Health Development Initiative (PHDI), Monrovia, Liberia

**Keywords:** Recovery, Reconstruction, Ecological, Transformation, Ebola, Preparedness, Resilience, Response, Africa

## Abstract

Rebuilding transformation strategies in post-Ebola epidemics in West Africa requires long-term surveillance and strengthening health system preparedness to disease outbreak. This paper assesses reconstruction efforts from socio-cultural, economic and ecological transformation response approaches and strategies in improving sustainable survivors and affected communities livelihood and wellbeing. A comprehensive approach is required in the recovery and rebuilding processes. Investing in rebuilding transformation requires fostering evidence-based and effective engaging new investors partnership strengthening, financing community-based programmes ownership, novel socio-economic innovations strategies and tools against the evolving and future Ebola epidemics. Thus, there should be improved community partnership, health and economic rebuilding programmes to address mistrust and care underutilization, poverty and care access inequity at all levels. Implementing effective post-Ebola national ‘One Health’ approach coupled with climate change mitigation and adaptations strategies is urgent public health needs aiming at improving the quality healthcare access, delivery trust and uptake in anticipation of EVD immunization program, productivity and emerging economy.

**Electronic supplementary material:**

The online version of this article (doi:10.1186/s40249-017-0278-2) contains supplementary material, which is available to authorized users.

## Multilingual abstracts

Please see Additional file 1 for translations of the abstract into the five official working languages of the United Nations.

## Introduction

Almost four decades since Ebola epidemic was first identified, we have learnt that prevention measures are better than emergency responses to infection with the Ebola virus that causes severe haemorrhages and multiple organs failure. In confronting the invisible Ebola virus in Africa, neither Ebola infection, nor Ebola Virus Disease (EVD) survivors breast milk and semen persistence, social stigmatization and disability occur in a vacuum [1, 2]. Lessons learnt from West African public health system have shown the unpreparedness of the national health systems toward EVD preparedness, prevention and containment [2, 3]. Revamping recovery and rebuilding of the local and national health systems including emergency response requires access to and use of medical and stockpile system network in tackling the existing patchy and sporadic supply chain procurement and management [1, 3]. Moreover, addressing the drawbacks of surveillance, detection and tracking systems of pathogen (s) transmission dynamics before the onset of potential threat and outbreak have not been achieved in most African countries [2, 4, 5]. The threats posed by EVD survivors and long-term latent asymptomatic reservoirs can be amplified by socio-behavioural and ecological risks factors as well as economic and epidemiological determinants [4, 6]. Thus, accelerating the recovery and rebuilding process in affected countries has immense potential benefits by positively influencing the local communities across Africa that outwitting the persistent pattern of infectious disease threats and outbreak burden is real. In addition, better understanding of risk factors should be invigorated by collaborative joint operational Ebola research and development agenda toward evidence-based decision making policies and programmes [2, 5–7]. Forecasting in knowing the nature of the EVD epidemic in a given African context, it is crucial that contextual, robust and sustainable recovery and reconstruction programmes and strategies are developed and implemented with sustainable financial, economic and policy management mechanisms. These could accelerate and sustain community engagement and resilience required for continuous social mobilization, health education and awareness, and promoting regulatory reforms that will enhance public health strengthening and standard interventional practices. Also, spring up local innovations in local and international preparedness and emergency response measures enhancements is needed in threats and epidemics capacity development and management from global health to local health-care workers and laboratory staff preventive and smart response strategies perspectives [4, 7, 8]. There is an urgent need for greater leadership commitment and investment efforts in integrated medical, psychosocial and economic recovery and reconstruction needs and programs. These include intensifying community engagement and resilience, citizenry awareness and education, efficient and sustainable financial and market development and benefits. Strengthening of EVD funding of recovery programmes and positive human socio-cultural behaviour will improve the national EVD recovery programmes such as Ebola national immunization, public health security and economic prosperity. Improving the lives and livelihood of the survivors and affected communities aiming at short and long-term individual, community and governments joint engagement and commitment into primary and secondary recovery and reconstruction projects and programmes performance and effectiveness. These include providing the enabling recovery and rebuilding environment (political, regulations and laws), national security and peace, assisting new investors and entrepreneurs, tax relief and cross-border trade incentives, reducing Ebola virus resurgence from survivors, survivors access to and use of psycho-social and care delivery counselling, enhanced schools or faith-based education curriculums and skills training including safe cultural burial practices at all levels, boosting employment and regain of income, facilitating access to financial support or loan, accelerating local/national disaster risk reduction and emergency response implementation and actions plans awareness.

Hence, this paper examined recovery and rebuilding programmes through socio-cultural behaviour, economic and ecological approaches and strategies in improving contextual, effective and sustainable post-EVD outbreak transformation era, assurance, trust and empowerment in early alertness and resilience culture amongst survivors, affected communities Africa and worldwide.

## Rebuilding effective national health systems and community-based programs

The variation in the West Africa Ebola outbreak impact provided new dimensions on the drawbacks and challenges of the health system care practice, coupled with potential survivors’ persistent disease transmission dynamics and spread. Strengthening the primary health systems, community engagement and social mobilization are crucial in mitigating the effectiveness and sustainability of first and second order impacts rebuilding and recovery programmes and activities in affected populations and Ebola survivors. These include community assessment of overall needs and priority, aligning needs and demand based on vertical and horizontal equitable support programmes from government and local private sector, International Monetary Fund (IMF) post-Ebola relief fund and United nations (UN)-global health security agenda [5, 6, 9, 10]. For example scaling up access to and uptake of sustainable recovery programmes including EVD national immunization coverage in improving maternal and child healthcare, family planning and breastfeeding, survivors counselling and psychosocial centers, early detection and diagnostic tools for prompt treatment of asymptomatic case(s) resulting from Ebola virus persistence in breast milk and semen of survivors in affected areas, microfinance support, policy and regulation reforms. Moreover, fostering local and foreign investment, local microfinance and entrepreneurship activities in fastening recovery and reconstruction programmes effectiveness are needed. Strategic rehabilitation and reconstruction policy and governance is needed to scale up access to equitable prevention service and justice, gender-based violence mitigation advocacy and contextual technical mentorship support. Nurturing, inspiring and empowering talents, local resilience and performance, and standard practices adherence initiatives is crucial for the overall healthy development and economic growth [1, 10, 11]. Foremost short and long-term commitment of governments and new stakeholders partnership and collaborative investment efforts should be focused on financial, socio-economic and educational recovery opportunities, tax relief and health insurance, agricultural development toward food security and sanitation programs; population access to flexible microfinance loan and grant applications for business development, youth-targeted skills empowerment and economic diversification (Table 1).

**Table 1:** Summary of post-Ebola survivors’ recovery and rebuilding phased programmes and activities

Overall Strategic plan	Recovery and rebuilding programmes and activities	First-order impact actions and strategies	second-order long term mitigation and response impact
Surveys assessment of recovery mechanisms and rebuilding activation.	- Public health and Community engagement and social mobilization - Harnessing needs and demand-based inclusive and equitable recovery activities capacity - Understanding the counter factors and challenges (existing realities, drivers and barriers) - Quantitative and qualitative data collection tools evaluation and standardization	- Strengthening national institutions and infrastructures support to tackling risk and Ebola persistence through national EVD immunization program - Training and capacity development including local staff and target groups, government and stakeholders - Positive community perception, knowledge, attitude and practice changes - Active psychosocial and welfare support services of survivors, orphans and child protection - Enabling economic and time-bound business plans and operations	- Improving local/national reforms, partnership and financial operational and management accountability - Continuous development of financial and labour market capabilities for community educational trust confidence and empowerment - Expanding emergency and community support systems and ownerships to other areas - Revamping entrepreneurship education and agricultural sector
Implementation and performance of interventions and changes metrics assessment	- More inclusiveness recovery and rebuilding response investment - Operational trading and financial recovery activities and structural measures - Implementation of protocols, SOPs, data and database security, quality control management systems - Timeliness and mutually beneficial community transformation options and productivity	- Data and information capturing, processing and analysis as well as reporting to stakeholders - Operational infrastructure services resources such as electricity, water, agriculture, security, telecommunications, environmental needs -Enhanced recovery plans and contingency measures approaches implementation for social cohesion and peace	- Tax relief and secured microfinance activities for private sector firms and import–export regulations - Revamping growth and job creation, rebuilding local economy subsidies - Integrated evidence and coordinated outputs - Consolidation of most potent investment and innovations
Outcomes/impact and specific innovations	- Achieving macroeconomic and healthy workforce development post-Ebola recovery agenda - Assessment of organisational impacts, planning, resilience and recovery mitigation information - Fostering post-Ebola disaster needs public-private partnerships (PPP) innovative responses - Timeframe recovery process, regulation and policy changes	- Addressing, evaluation, learning and reporting. tracking adaptations and strategic implementation - Benefits and fitness for reoccupation of target populations (accommodation, training, good and services supply chain, food and entertainment) - Increase collaborative resources by forming partnerships and networks - Expanding early warning, forecasting and improved WASH in affected communities	- Investment in regaining jobs, market growth, micro and macro-economic trade-off, as well health insurance - Community projects partnership and ownership resilience and investment - Community projects/programs ownerships including R&D - Long-term health monitoring and social protection of survivors, orphans and affected communities
Monitoring and evaluation activities	- Examining the extent and nature approaches of second order impact implementation and sustainability	- Rebuilding trust, engagement and resilience markets growth - Efficiency-driven participation endeavours	- Recommendations in decisions making policy, innovations and collective responsibilities

Strategic global post-EVD epidemics assistance in recovery and rebuilding programmes and activities institutionalization is imperative in improving care workforce and health commodities, access to and use of diagnostics and care delivery packages to remote rural settings [6, 8, 12]. Substantial success rely on improving information communication, training of field workers, care-providers and epidemiologists, leadership management and accountability, quality standards at local and national levels [3, 7, 8]. Setting up evidence-based comprehensive phased approaches such as first and second-order impact agenda is needed for the implementation of quality management policies, process and tools in rehabilitating the affected populations and survivors as well as reconstruction of the affected communities’ socio-economic regains supported by the community and all stakeholders [1, 13, 14] (Table 1).

Although recovery is a complex process, community engagement and social mobilization activities play an important role in developing and implementing operational and transparent evidence-based rebuilding and recovery programmes [8, 9]. Moreover, community perceptions and vision of decision makers in building comprehensive and integrated evidence-based approaches and strategies is crucial in strengthening emergency local and national health systems, psychosocial and economic recovery and management capacities imperative to public peace and security for partnership commitment and investment [8, 9, 12]. Understanding the local determinants can also contribute to increasing the frequency of public health needs, programs and social services delivery amongst communities and populations who are at greater risk or disease outbreak disaster. Response policies and programs should be rooted within evidence-based frameworks and standards practices [8, 12]. This should include a multidisciplinary training and workshops, interactive discussions, group work and individual/collective projects in answering some key socio-cultural and health issues and players [8–10]. What and how do we communicate with each other? How do we plan together? Who is responsible for doing what and when? Thus, implementation of effective and efficient community capacity to responses are of greater opportunities in establishing effective community sensitization activities using the survivors. Fostering accessibility of care centers and laboratory support services should be primarily based on proactive community health workers and volunteers capacity building and training in early risk communication, safe isolation, quarantine and contact tracing, safe burial practices and preventative measures adherence [10–12]. Strengthening the health systems operational preparedness and readiness plans is needed in improving availability of stockpile and supply chain management system as well as sustainable mechanisms and maximizing the advantages of direct technical assistance and guidance from stakeholders toward community recovery projects/programs ownership at all levels [15, 16, 17]. Establishing emergency preparedness plans and coordinated response units and activities is needed by implementing disaster risk reduction and National Framework for Health Emergency Management (NFHEM)with adequate coordination and planning mechanisms that can be mobilized during emergencies or disease outbreak in line with Sendai declaration [7]. The role of communication education in mediating the intersections among these variables is critical in its contribution to decreasing the spread of EVD and mitigating the impact on individuals and the education system [8, 11, 17, 18]. For example, surveillance and information sharing increased at the neighbouring borders districts of Guinea, Liberia and Sierra Leone with intense EVD transmission with Côte d’Ivoire, Mali and Senegal [1, 9]. Such an innovative system supports the reinforcement of extra-curricular and non-formal education interventions for adults and drop out, door to door household campaigns as well as aged population in the community [5, 13, 17]. Health education and promotion should be tailored towards culturally-sensitive and age appropriate curricula, taught or disseminated by well-trained teachers/preachers and/or community trained health promoters, community workers or mobilizers with practical demonstrations of opportunities and gains on individual, family and community at large positive transformations and impact [3, 13–15, 19] (Table 1).

As the root causes to vulnerability are socio-cultural, economic and poverty related in nature, the educational system should be transformed in a way to prevent EVD and other future epidemics [7, 16, 17]. Social mobilization and education could properly resource and support schools and community to ensure quality programmes and measurement of meaningful results with proven tools and methodologies being easily adapted to community-specific contexts including scaling up cultural and behavioural preventive measures and tools [1, 8, 16]. Lessons learnt coupled with technical assistance could contribute to an integrated community preparedness projects and knowledge-base that features cross-fertilization and improvement in local capacity and response delivery [16–18]. The African educational system must be innovative to local realities in order to inform, mobilize and reinforce community effective planning, early warning alerts indicators and prompt efficient response to risk and epidemic or disaster. Monitoring and evaluation of socio-cultural and ecological factors in these communities is core in improving quality evidence-based decision-making and care delivery interventions delivery on persisting survivors neurological, musculoskeletal and ocular effects for significant socio-cultural transformation and development at all levels [1, 4, 7, 9, 16, 20]. Such collective behaviour changes could lead to improvement in health seeking attitudes, cultural emancipation in safe burials practices, compliance to standard protective measures and quality health outcomes toward advanced health care system benefits regionally [16–18].

## Leveraging on citizenry rebuilding resilience and empowerment methods

The exchange of experience on social norms and collective behaviour and their impact on both driving infection and influencing prevention planning and management efforts should be encouraged. Also, promoting social environment of understanding and tolerance that enables learning and transformation to more positive behaviour adaptations should be encouraged [2, 13, 21–23]. To optimize such goals, community soft environment awareness including drama, theatre shows, media, poems and others projects should be incorporated into the social media and education programs [13, 23, 24]. Leveraging on local apolitical champions and international leaders to communicate vital measures and instructions are necessary [1, 4, 10]. Therefore, the need to create local based risk reduction and emergency responses and interventions mechanisms where actors are free to reflect critically on their historical beliefs and traditions, is necessary in implanting the enabling working environment [12, 15]. Likewise, fostering accessibility to health services including protective measures uptake, provision of guidance and assistance, community mobilizers/watch teams, workshops, groups discussions and campaigns, sensitization on safe burial and transformation programmes are important [2, 19] (Table 1).

Fostering efficient formal and informal skills and knowledge development and professional improvements in health, management, agriculture production and food security (fertilizers, machinery) is paramount in achieving long-term hope and increasing household cash crops and productivity [19]. This will increase the overall local food auto-sufficiency and economy, by expanding the structure of national and regional economic expectations and growth [10].

While post-Ebola recovery and rebuilding actions plans also require building integrated capacity within local and neighbourhoods context, adapting proactive and effective to the new environmental and culture, targeted resilient mass media and social media information dissemination of proven solutions in improving awareness and empowerment is imperative in regaining community trust and speeding civic accountability [9, 13, 25, 26].

## Transforming contextual behavioural and attitudes information dissemination strategies

Historically, African communities have strong traditional practices for dialogue and information dissemination through local means including drums, chief messengers, oral history and anecdotes and other cultural ways of keeping with ancestors for promoting mobilization, communication, caring for the sick and safe burial practices [8, 14]. Social cohesion, inclusive growth and community compassionate support in supporting the shared social, cultural and religious values and realities are the foundation to mitigate infectious diseases risks by finding acceptable and resilient preventative ways [8, 27, 28]. It is difficult to ascertain what technique or combination of tools and approaches could guarantee that Ebola would neither resurge nor spread in the future [29, 30]. Hence, the needs for robust and innovative health education curricula and interventions implementations in schools and faith groups (childhood, adults women and elderly) to boost understanding of cultural norms, environmental and socio-economic contexts to risk and epidemics. Particularly, empowering survivors and citizenry with new knowledge and skills are necessary in adopting positive attitudes and practices against evolving risk and epidemics. These should emphasize on increasing contextual awareness and vigilance, counselling on sexual and reproductive health and family planning, use of condom, improved HIV/EVD screening programs, enhanced access to water, sanitation and hygiene which should be constantly reinforced based on sound understanding and freedom of choice including Africa oral history and anecdotes [5, 8, 29, 31]. Furthermore, the importance of innovative evidence-based knowledge and practices of socio-cultural and behavioural practices in EVD and other epidemic prevention, care and mental support and counselling should be entrenched in African traditions and story-telling [19, 32, 33]. Current efforts in community mobilization campaigns and intense communication and dialogue with communities have recorded much progress on positive attitudes to health seeking and traditional practices in reducing future EVD incidence and fatality in West Africa epicentres, DR Congo and other proxy-countries [3, 8, 22, 28, 34]. Promotion of preventive measures and health education among adolescents and youngsters should be expanded at all levels. Simple and reliable multistage technical skills development and training empowerment, drama and theatre performances by local eloquent heroes such as entrepreneurs, footballers, musicians, comedians and other stakeholders could be crucial in rebuilding trust and hopes in cultural, social and behavioural recovery and reintegration [8, 35–38]. Collaborative community-driven projects ownership and active social accountability and transparency are needed in rolling out national EVD immunization program which is crucial positive step including population-based awareness, blood, semen and breast-milk testing assessment for survivors, public health strengthening for socio-economic transformation and growth [8, 14, 19, 39].

## Conclusion

Although Ebola epidemics 2014–2016 was an immense impediment and challenges in economic development and growth in West Africa and Africa in general, investing at promoting rapid recovery and reconstruction programs is crucial. Better investment including local private sector collaborative and resources allocation in contextual and effective impact mitigation approaches, rebuilding and strengthening health systems capabilities are vital against evolving outbreaks consequences. Understanding and monitoring the social and behavioural dimensions needs to be active and sustained to guarantee the effectiveness of survivors’ resilience support systems. Promoting innovative and inclusive community-based multi-disciplinary and inter-sectoral entrepreneurship approaches should be enhanced. Social responsibility and solidarity, health promotion, counselling on positive socio-behavioural, health and cultural attitudes and behavioural changes are crucial in developing trust and confidence in national/regional reconstruction. Sustainable community-based partnership is vital in fostering community engagement and education, and shared values. Implementing local/national ‘One Health’ approach in early risk communication, timely resilience and emergency response projects/programmes ownership are needed to keep pace with positive socio-cultural and behavioural mitigation approaches and shared economic benefits across Africa.

## Additional file

Additional file 1: Multilingual abstracts in the five official working languages of the United Nations. (PDF 639 kb)

## Abbreviations

EVD: Ebola virus disease
HIV: Human immune deficiency virus
IMF: The International Monetary Fund
SDG: Sustainable Development Goals
UHC: Universal Health Coverage
UN: The United Nations
