# Supplementary material for: Rebuilding transformation strategies in post-Ebola epidemics in Africa
Source: Infect Dis Poverty. 2017 May 10;6:71. doi: 10.1186/s40249-017-0278-2 (PMC5424336; doi:10.1186/s40249-017-0278-2)
Supplement: Additional file 1: — Multilingual abstracts in the five official working languages of the United Nations. (PDF 639 kb) [file 40249_2017_278_MOESM1_ESM.pdf]

Translation of the abstract into the five official working languages of the United Nations

إعادة بناء استراتيجيات التحول في ما بعد أوبئة فيروس الإيبولا في أفريقيا

إرنست تامبو، كريسيس ف تشينجو، تشيديبيري ي أوجوو، إساتا وري، جينيتا ك جونسون، جين ي. نجوجانج

ملخص
إعادة بناء استراتيجيات التحول في ما بعد أوبئة الايبولا في غرب أفريقيا يتطلب مراقبة طويلة الأجل وجاهزية نظام تعزيز الصحة عند ظهور المرض. تقيم هذه الورقة جهود إعادة التنظيم من نهج استجابة التحول الاجتماعي والثقافي والاقتصادي والبيئي واستراتيجيات تحسين سبل العيش والرفاه المستدامة للناجين والمجتمعات المتضررة. المطلوب نهج شامل في عمليات الإنعاش وإعادة البناء. الاستثمار في تحويل إعادة التنظيم يتطلب تعزيز استراتيجيات الإشراك الدليلي والفعال للمستثمرين الجدد لإيجاد شراكة فعالة، وتمويل البرامج المجتمعية واستراتيجيات الابتكارات الاجتماعية والاقتصادية ضد أوبئة الايبولا قريبة الظهور والمستقبلية. وبالتالي، ينبغي تحسين الشراكة المجتمعية والصحية وبرامج إعادة البناء الاقتصادي لمعالجة الفقر وعدم الإنصاف في التوصل إلى خدمات على جميع المستويات. تنفيذ نهج صحي واحد وطني فعال لما بعد الايبولا واستراتيجيات لتخفيف تغير المناخ إلى جانب البرنامج الوطني للتحصين من مرض فيروس الإيبولا والاحتياجات الصحية العامة العاجلة التي تهدف إلى تحسين الوصول إلى جودة الرعاية الصحية، وفهم الاحتاياجات والإنتاجية والاقتصادات الناشئة.

Translated from English version into Arabic by Mahmoud Sami, through

TRANSLATORS
WITHOUT BORDERS

非洲后埃博拉时代重建转化策略

Ernest Tambo, Chryseis F Chengho, Chidiebere E Ugwu, Isatta Wurie, Jeannetta K. Jonhson, Jeanne Y. Ngogang

摘要
在西非埃博拉疫情中重建转化战略需要长期监测并加强卫生系统储备能量以应对疾病暴发。本文评估了社会文化、经济和生态转化应对方法和策略在改善幸存者和受影响社区的生计和福祉方面的重建行为。需要综合的方法来进行恢复和重建。重建转化投资需要加强组建基于证据并有效的投资伙伴关系、资助基于社区的项目所有权、新颖的社会经济创新战略和工具以应对不断演变和未知的埃博拉疫情。因此，应当改善社区伙伴关系，优化健康和经济重建方案，以解决各阶层的贫困和保健机会不均等问题。目前，公共卫生领域迫切要求实施有效的后埃博拉全国“同一健康”方式、减缓气候变化的适应战略和国家 EVD 免疫计划，目的在于提高医疗服务质量、增强交付体验、促进生产力和新兴经济的发展。

Translated from English version into Chinese by Jin Chen, edited by Yang Pin, through

CDC CHINESE CENTER FOR DISEASE CONTROL AND PREVENTION
NATIONAL INSTITUTE OF PARASITIC DISEASES

Reconstitution de stratégies de transformation suite à l’épidémie d’Ebola en Afrique

Ernest Tambo, Chryseis F Chengho, Chidiebere E Ugwu, Isatta Wurie, Jeannetta K. Jonhson, Jeanne Y. Ngogang

Résumé

La reconstitution de stratégies de transformation suite à l'épidémie d'Ebola en Afrique de l'Ouest nécessite une surveillance à long terme et le renforcement de la préparation du système de santé aux épidémies. Le présent article évalue les efforts de reconstruction consentis dans le cadre d'approches et de stratégies de transformation socio-culturelle, économique et écologique permettant d'améliorer la pérennité des moyens de subsistance et du bien-être des survivants et des communautés affectées. Une approche exhaustive est requise pour les processus de récupération et de reconstruction. L'investissement dans la reconstitution de stratégies de transformation exige la promotion d'un engagement reposant sur des données factuelles et efficace, le renforcement de la conclusion de nouveaux partenariats d'investissement, le financement de l'appropriation de programmes communautaires ainsi que de nouveaux outils et stratégies socio-économiques innovants permettant de lutter contre l'évolution et les futures épidémies d'Ebola. Il convient donc d'améliorer le partenariat communautaire et les programmes de reconstruction en termes de santé et d'économie afin de lutter contre la pauvreté et les inégalités d'accès aux soins à tous les niveaux. La mise en œuvre d'une approche « Une seule santé » nationale et efficace suite à l'épidémie d'Ebola ainsi que de stratégies d'adaptation aux changements climatiques associée à l'exécution d'un programme national de vaccination contre le virus Ebola constituent des priorités de santé publique qui permettront d'améliorer l'accès à des soins de santé de qualité, l'adoption de la prestation, la productivité ainsi que l'économie émergente.

Translated from English version into French by eric ragu, through

TRANSLATORS
WITHOUT BORDERS

Стратегии преобразований для восстановления в обстановке после эпидемий Эбола (пост-Эбола) в Африке

Ernest Tambo, Chryseis F Chengho, Chidiebere E Ugwu, Isatta Wurie, Jeannetta K. Jonhson, Jeanne Y. Ngogang

Аннотация

Стратегии преобразований для восстановления в обстановке после эпидемий Эбола (пост-Эбола) в Западной Африке требуют длительного наблюдения, а также повышения уровня подготовленности системы здравоохранения к эпидемической вспышке болезни. В этой статье оценивают усилия по восстановлению, начиная с оперативных подходов и стратегий в виде социально-культурных, экономических и экологических преобразований для улучшения условий жизни. Целью таких мер является долговременная работа по обеспечению средств к существованию и поддержке благополучия у лиц, выживших после лихорадки Эбола, и членов пострадавших сообществ. Необходим комплексный подход в процессах выздоровления и восстановления. Инвестиции в преобразования для восстановления требуют стимулирования научно-обоснованного и эффективного участия новых инвесторов, укрепления их сотрудничества, финансирования территориальных программ собственности, новых социально-экономических инновационных стратегий и мер, направленных на борьбу с формирующимися и будущими эпидемиями, вызванными вирусом Эбола. В связи с этим следует улучшить программы восстановления партнерства в области общественного здравоохранения и экономики с целью решения проблемы бедности и отсутствия равных возможностей доступа для оказания медицинской помощи на всех уровнях. Внедрение эффективного национального подхода «Всеобщее здоровье» ('One Health'),

а также стратегии адаптации и смягчения влияния климатических изменений совместно с национальной программой иммунизации против заболевания, вызванного вирусом Эбола (Ebola virus disease, EVD), являются неотложными задачами и потребностями общественного здравоохранения. Эти мероприятия нацелены на улучшение доступа для оказания качественной медицинской помощи, уровня использования гуманитарных поставок, повышение производительности и поддержку развивающейся экономики.

Translated from English version into Russian by Ann Nosova, through

Reformular las estrategias de transformación en las epidemias pos-ébola en África

Ernest Tambo, Chryseis F Chengho, Chidiebere E Ugwu, Isatta Wurie, Jeannetta K. Jonhson, Jeanne Y. Ngogang

Reseña

Reformular las estrategias de transformación en las epidemias pos-ébola en África Occidental requiere vigilar y fortalecer la preparación del sistema de salud para el brote de la enfermedad. Este trabajo evalúa los esfuerzos de reconstrucción desde enfoques y estrategias de respuesta de transformación socio-culturales, económicas y ecológicas para mejorar el modo de vida y bienestar sostenible de los sobrevivientes y de las comunidades afectadas. Se requiere un enfoque amplio en los procesos de recuperación y reconstrucción. Invertir en reformular la transformación requiere el fortalecimiento de nuevas asociaciones con inversores que sean involucrados en forma efectiva y sobre la base de pruebas y financiar la titularidad de programas basados en la comunidad y las herramientas y estrategias de innovaciones socio-económicas noveles contra la evolución y futuras epidemias del Ébola. Por lo tanto, debe haber una mejor colaboración de la comunidad y programas de reconstrucción económica y de la salud para abordar la pobreza y la inequidad en el acceso al cuidado en todos los niveles. Implementar un enfoque efectivo de "una salud" a nivel nacional y estrategias de adaptación de mitigación unidas al programa de inmunización contra la EVE son necesidades de salud pública urgentes que aspiran a mejorar el acceso al cuidado de la salud de calidad, el uso del parto, la productividad y la economía emergente.

Translated from English version into Spanish by LidiaN, through
